# Factors Affecting Long-Term Outcomes for Patients with Inflammatory Bowel Disease—A Cross-Sectional Design

**DOI:** 10.3390/nursrep15070231

**Published:** 2025-06-25

**Authors:** Ulrica Lovén Wickman

**Affiliations:** Department of Health and Caring Sciences, Linnaeus University, 392 31 Kalmar, Sweden; ulrica.lovenwickman@lnu.se; Tel.: +46-070-2231768

**Keywords:** coping, health-literacy, health-related quality of life, inflammatory bowel disease, long-term outcomes, nursing, self-care strategies, social support

## Abstract

**Background:** Symptoms of and treatments for inflammatory bowel disease have an impact on patients’ health-related quality of life and result in a need for self-care strategies. Little is known about factors affecting long-term outcomes and the types of coping strategies used by adult patients with inflammatory bowel disease to better cope with their chronic illness. **Objective:** This study aims to explore coping strategies, social support, and health-related quality of life and describe factors affecting long-term outcomes for patients with inflammatory bowel disease. **Methods**: A cross-sectional design was used, with a consecutive sample of 206 patients with inflammatory bowel disease who were recruited at three gastroenterology clinics in Sweden and given surveys consisting of patient characteristics, the Brief COPE, and a social support questionnaire. Descriptive statistics were used to analyze the data. This study was guided by Strengthening the Reporting of Observational Studies in Epidemiology (STROBE) guidelines. **Results**: The sample was 53% women and included 206 patients with a median age of 48 years. The coping mechanisms often used were active coping methods (problem-focused). Most of the patients had someone special by whom they felt supported (89%). Gender differences were shown for emotional support and whether the patients had someone they felt close to. According to the findings, less bowel interfering and social support correlated with higher well-being. Worry was associated with giving up, symptom burden, and less bowel interfering. No significant correlations were shown for symptom burden and social support. **Conclusions**: Social support, especially from someone at home or offering comfort, was positively linked to well-being. Active, problem-focused coping was common and associated with better outcomes. Notably, no direct link was found between symptom burden and coping or support, underscoring the complexity of these relationships. These findings emphasize the need for psychosocial interventions to enhance coping and support, ultimately improving health-related quality of life in IBD.

## 1. Background

Crohn’s disease (CD) and ulcerative colitis (UC) are grouped under the name inflammatory bowel disease (IBD), and as the name reveals, the pathology is due to inflammation in the gastrointestinal tract [1]. The treatment is lifelong and complex, including several different pharmaceutical groups and sometimes surgery [2]. The debilitating symptoms, such as abdominal pain and bloody diarrhea, and the need for healthcare and contact with healthcare professionals are lifelong. The symptoms affect functioning in daily life and perceive health-related quality of life. Worries and concerns can affect this perception [3]; commonly experienced symptoms with IBD are both physical and psychological [4,5]. Symptoms of IBD may have various causes, and medical treatments may be used to target the symptoms and affect the underlying mechanisms. If symptoms persist, the patient must find self-care strategies to help them handle such situations [5]. UC and CD differ according to the location and extent of inflammation in the gastrointestinal tract, affecting the symptom experience [6]. Several studies have explored the life situation for patients with IBD and their family members [7,8]. In one review, the results showed that patients described living in secrecy, including having an invisible illness and not being believed, due to a lack of public awareness. Having an invisible illness raised questions from friends and family, which made the patients feel that they were not believed [7].

Patients with IBD need to be motivated and engaged in their care, and self-care is important for successful treatment. The value of self-care is in working to be healthy and paying attention to caring for oneself and one’s feelings, aimed at reducing symptoms. Self-care also allows the patient to get involved with others and ask for help [9,10]. There is an awareness that patients may vary in their motivation to enact behavior change according to the Transtheoretical Model of Change [11]. Patients with IBD actively participate in self-care to manage and adapt to their symptoms. This involves recognizing and handling symptoms, planning daily activities, and exploring new management options. Daily self-care practices among patients with IBD include adherence to medication, making dietary adjustments, and ensuring urgent access to a toilet, all of which significantly impact their daily functioning [12]. A protocol paper outlines the design and methodology of a planned study referring to self-care in patients affected by IBD and caregiver contribution to self-care [13].

Variability in performing health behaviors is common and influenced by multiple individual factors, including self-efficacy (i.e., confidence) for performing specific tasks. Self-efficacy is linked to improved disease adjustment, as individuals feel empowered to actively manage their condition, adhere to treatment plans, and effectively cope with the challenges posed by the disease [14]. Higher self-efficacy is shown to be associated with lower daily IBD burden, and the relationship between patient activation and IBD burden is mediated by self-efficacy [15].

Coping is a widely used term in healthcare, and its significance has varied over time [16,17]. The literature encompasses a range of psychological and behavioral strategies patients employ to address and adapt to the demands of stressful situations. These strategies may vary by personality, life experiences, and stressors. Coping mechanisms for individuals with IBD may need to adapt to the unpredictable nature of the disease, including periods of remission and flare-ups. Flexibility in coping strategies and learning to manage symptoms may increase disease activity [18]. Coping mechanisms refer to problem-focused, emotion-focused, and avoidant coping. Problem-focused coping involves tackling the stressor directly, seeking practical solutions, and taking action to mitigate the source of stress. Emotion-focused coping entails managing the emotional distress associated with the stressor, often through strategies like seeking social support, practicing mindfulness, or reframing one’s perspective, and the wide range of work-related physical and psychosocial impairments affecting patients with IBD [19].

Social support involves aid, encouragement, and understanding within a social network. The network extends beyond familial ties to include friends, colleagues, and community members. These types of social support include emotional support and the expression of empathy, love, and companionship, which provide patients with a sense of belonging and emotional reassurance. The effect of social support contributes to a patient’s overall sense of well-being [20].

Health literacy can include a variety of individual skills, both basic and interactive, as well as critical. This means that a patient understands the basics of examinations, prevention, diagnosis, and treatment. Furthermore, it is about the patient’s ability to analyze and evaluate health information and develop health-promoting problem-solving and action abilities [21]. One review expresses the role of education and knowledge, recommending that patients be guided to the official websites of healthcare and patient support organizations. Evidence-based educational portals can support patients in better understanding prescribed medications, and participation in educational programs can enhance treatment adherence and health outcomes. Healthcare professionals can improve patients’ adherence to knowledge and health outcomes through communicative strategies and regular follow-up [22].

Health-related quality of life among patients with IBD is explored in several studies [23,24]. Health is “a state of complete physical, mental, and social well-being and not merely the absence of disease or infirmity” [25]. Other definitions of health focus more on a person’s functioning level and expectations and how these may differ based on social well-being standards. The definition of health formulated by the WHO is considered sufficient to deal with the new challenges in healthcare [26]. Many factors influence health-related quality of life, including physical function, mental or psychological status, and engagement in normative social interactions. Also, levels of independence affect quality of life [27]. Factors influencing the quality of life in patients with IBD are, for example, chronic pain, diet, physical activity, and psychological factors like depression, anxiety, and stress symptoms [28].

Research within self-care strategies among patients is needed to cope with IBD. The knowledge gap relates to how factors influence long-term outcomes for patients with IBD. Examining coping strategies, social support, and health-related quality of life provides a nuanced understanding of the processes that influence long-term outcomes in patients with IBD. Social support may serve as a moderating factor in the relationship between disease-related stress and coping, thereby enhancing psychological resilience. Integrating these constructs enables the development of future interventions aimed at improving both psychological well-being and disease self-management. Understanding the patient’s subjective experiences can improve the healthcare professional’s ability to support and increase the patient’s self-care and health-related quality of life. This study aims to explore and discuss factors affecting long-term outcomes for patients with IBD, focusing on factors of social support, coping mechanisms, and health-related quality of life.

## 2. Methods

### 2.1. Design

A cross-sectional design with a consecutive sample of patients with IBD was used. This study was guided by The Strengthening the Reporting of Observational Studies in Epidemiology (STROBE) guidelines [29].

### 2.2. Sample and Setting

Adult patients from three gastroenterology clinics in Sweden were asked to participate. Patients were recruited into the project while visiting the gastroenterological clinic. The inclusion criteria were adult patients with confirmed IBD, and the questionnaires were only handed out to patients with either CD or UC. However, six patients did not choose one of the two options; all six were included in the UC group. Exclusion criteria were lacking knowledge of the Swedish language. Physicians and nurses at the respective clinics recruited patients and distributed the questionnaires to the ones that chose to participate. In total, 206 patients living with IBD completed the questionnaires (46% response rate).

### 2.3. Data Collection and Measurement

Study information was provided by nurses or physicians at each gastroenterology clinic through an information letter that included study objectives and descriptions, what volunteering entailed, and information about the ability to withdraw at any time. The time frame for data collection was from December 2015 to May 2017.

#### 2.3.1. The Brief Cope

The Brief Cope is a 28-item, patient-reported, widely used coping questionnaire measuring patients’ subjective experience living with IBD. The Brief Cope is designed for ease of use in research and clinical settings. This instrument identifies various coping strategies, including problem-focused, emotion-focused, and avoidant coping approaches. The items include self-distraction, active coping, denial, substance use, use of emotional support, use of instrumental support, behavioral disengagement, venting, positive reframing, planning, humor, acceptance, religion, and self-blame [30]. The Brief Cope developed as a condensed version of the original [31], which was theoretically derived based on various models of coping. While validation studies in Swedish populations are limited, studies using the Swedish version have generally reported Cronbach’s alphas ranging from 0.58 to 0.92 for the Brief COPE [32]. One study validated the scale among esports athletes and found the following means and standard deviations for each sub-scale: problem-focused—2.47 (0.63); emotion-focused—2.23 (0.49); and avoidant coping—1.64 (0.45) [33] (Table 1).

#### 2.3.2. A Modified Form of the Availability and Attachment Scale

A modified abbreviated form of the Availability and attachment scale consisting of the first 7 items was used to assess social support. The scale ranges of the responses best reflect the social support available to the participant when it comes to people in their surroundings. The abbreviated version has acceptable reliability but slightly lower than the complete version [34]. However, a modified form was used in this study (Table 2).

#### 2.3.3. The Short Health Scale

The Short Health Scale (SHS) was used to assess HRQOL. It is made up of four questions where the patient grades the answer on a scale from 0 to 100, measured in millimeters. The four questions cover the patient’s symptom burden, social function, disease-related worry, and sense of general well-being [35]. SHS has been validated for both CD and UC [30].

### 2.4. Statistical Analysis

Descriptive statistics were used to present patient characteristics, social support, coping strategies, and health-related quality of life. Data are expressed as the mean and standard deviation, median and range, or frequency and percentage. Spearman’s correlation was applied to explore bivariate associations between patient characteristics, coping, social support, and health-related quality of life. To explore differences between groups, the Mann–Whitney U test was used for continuous variables, such as age and the duration of IBD. The Chi-square test was used for categorical variables, such as sex, diagnosis, and surgery. The significance level was set to *p* < 0.05. All statistical testing was conducted using IBM SPSS Statistics Version 29.0.

## 3. Results

### 3.1. Patient Characteristics

Descriptive analysis was used to summarize the demographic characteristics of the population. The majority of patients had UC (62.6%), and the median time since diagnosis was 12 (0–50) years. There were no significant differences between the different groups of IBD regarding patient characteristics apart from having gone through surgical procedures related to their bowel disease, which were more common in patients with CD (X^2^ (1, N = 206) = 48.719, *p* < 0.001) (Table 3).

### 3.2. Social Support

Among both men and women, most of the patients had someone special by whom they felt supported and said that there was someone special for them to share their innermost feelings with when they felt happy (n = 184, 89%). Most said that someone holds them for support or comfort (82%). Most also said that at home, someone else appreciates what they do for them (n = 160, 78%). For the number of people they know and have contact with who have the same interests as them, many patients said they had one to five persons (n = 89, 64%) and fewer said they had more than five friends (n = 100, 48%)

### 3.3. Health-Related Quality of Life

**Figure 1:**
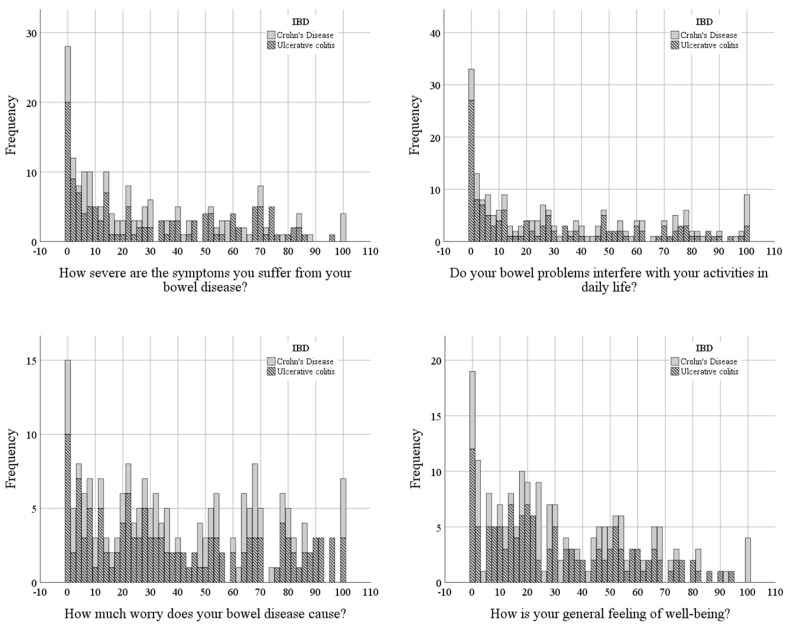
Visualization of the results from the Short Health Scale. Observe the different scales on the Y axis. Presented in a score range of 0–100. 0 = no symptoms, no interference with daily function, no disease-related worry, and a high sense of well-being. 100 = Very severe symptoms, very high degree of interference with daily life, constant disease-related worry, and a dreadful sense of well-being.

The answer rate for both groups of IBD was 100%. Cronbach’s α = 0.871 was shown for the SHS; however, examination of the median values was performed according to the four measured dimensions in the group of UC compared to CD (Table 4; Figure 1).

### 3.4. Coping Strategies Among Patients with IBD 

Cronbach’s α = 0.748 for the Brief COPE. Active coping (problem-focused), trying to come up with a strategy about what to do (mean = 2.78 ± 0.86) and taking action to try to make the situation better (problem-focused) (mean = 2.76 ± 0.87), and learning to live with the chronic disease (emotion-focused) (mean 2.95 ± 0.82) were the most frequently used coping strategies. However, substance use was the least frequently used strategy by the participants (1.14 ± 0.39). The top 10 results are presented (Table 5).

### 3.5. Associations Between Patient Characteristics, Coping, Social Support, and Health-Related Quality of Life

Gender differences were shown to be significant according to emotional support, whether the patients had someone they felt close to, and how many people they know (rho 0.204, 0.168, and 0.199, *p* < 0.05, respectively).

Coping activities and social support were significantly associated with health-related quality of life. Less bowel interfering correlated with higher well-being (0.607, *p* < 0.001). Social support, whether they had someone at home and someone to hold and comfort them, was positively associated with well-being (rho = 0.182 and 0.169, *p* < 0.05, respectively). Worry was associated with giving up (rho 0.183, *p* = 0.011), symptom burden, and less bowel interfering (rho 0.626 and 0.757, *p* < 0.05, respectively). No significant correlations were shown for symptom burden in the SHS, coping, and social support. Significant correlations for coping and well-being are shown in Table 6.

## 4. Discussion

This cross-sectional study explored coping, social support, and health-related quality of life in patients with IBD. The discussion will focus on factors affecting long-term outcomes for patients with IBD. When changing health behaviors, several elements, such as communication barriers, a lack of motivation, and structural constraints, can interfere with self-care. Factors influencing self-care including experience, skill, motivation, culture, confidence, habits, function, cognition, support from others, and access to care are described. Factors also include coping, health literacy, and adherence to treatment. The Health belief model explains why some individuals are more likely to engage in preventive health behaviors [36]. It is well known that symptoms motivate patients to engage in self-care, and interpreting symptoms is viewed as a self-care skill that develops over time when having a chronic disease [37].

The results express that active coping (problem-focused coping) is a common strategy for patients with IBD. The prevalence of coping in this study was highest for active coping strategies. When using active coping strategies, individuals act directly to solve problems and address stressors [18]. Earlier studies refer to emotion-focused coping being associated with worse psychological outcomes, and the effect of problem-focused coping was less consistently associated with better psychological outcomes [38]. A review has analyzed self-management interventions for patients with IBD. Interventions included education programs, digital tools, behavioral therapies, and multidisciplinary care models. Most interventions aimed to improve disease knowledge, medication adherence, symptom monitoring, and psychological well-being. Problem-focused coping strategies were often emphasized, such as goal setting, action planning, and communication with healthcare professionals. The review found positive effects on quality of life, self-efficacy, and reduction in disease activity [39].

The predominance of active coping (problem-focused coping) strategies among patients with IBD in this study may reflect a greater sense of agency and self-efficacy in managing their condition. Adults who have lived with IBD for a longer duration or who are currently in clinical remission may have developed more structured and proactive approaches to disease management. These individuals will likely have accumulated experiential knowledge and may feel more confident in navigating symptoms, adhering to treatment, and contacting healthcare professionals. Moreover, whether through formal programs or informal learning, health education could enhance their ability to engage in active coping (problem-focused coping). Access to specialist IBD nurses, gastroenterologists, and multidisciplinary care teams plays a role by providing guidance and emotional support. Additionally, adult patients may benefit from greater autonomy in decision-making and more consistent access to healthcare resources, which can reinforce problem-solving behaviors. Exploring how these contextual and individual factors interact could provide valuable insights for designing interventions supporting coping across different disease stages.

An important aspect highlights gender differences according to emotional support and whether the patients had someone they felt close to. Another study suggests that gender-specific variations in treatment and adherence can enhance disease management and promote a more individualized treatment approach [40]. IBD is a complex, chronic condition that creates uncomfortable and challenging situations. Individuals with IBD often avoid social interactions to prevent awkward situations [41]. Patients with IBD often experience significant psychological distress due to loneliness. Research indicates that 72% of patients with IBD face social isolation [42]. Avoidance of social activities can impact patients, resulting in increased loneliness and reduced social cohesion [12]. Gender differences in patients with IBD encompass the age of onset, disease location, and the prevalence of extraintestinal manifestations. However, no significant differences in the therapeutic management of IBD have been observed between male and female patients [43].

The results showed that most of the patients had someone special by whom they felt supported and felt that there was someone to share their innermost feelings with. High levels of social support can help to minimize stress, provide a buffer against psychological distress, and enhance self-care management [44]. The effect of social support contributes to a patient’s overall sense of well-being. Successful self-care management interventions need to leverage the support of one’s social network and adapt interventions to develop interventions to support patients with IBD [20].

A wide range of self-care activities influence daily life for patients with IBD intending to enhance their good health-related quality of life [12]. Variability in performing health behaviors is common and influenced by multiple individual factors. Several factors interact to enhance health-related quality of life, with self-care activities to handle the symptoms. One study expresses variability in both conditional health literacy and digital health literacy among patients with IBD. Patients with higher health literacy were more likely to understand and evaluate health information, use digital tools (e.g., apps, portals) to manage their condition, and communicate effectively with healthcare professionals. Lower literacy levels were associated with older age, lower education, and limited digital access. Considering patients’ literacy levels is important to ensure equitable access to information and care [45].

**Figure 2:**
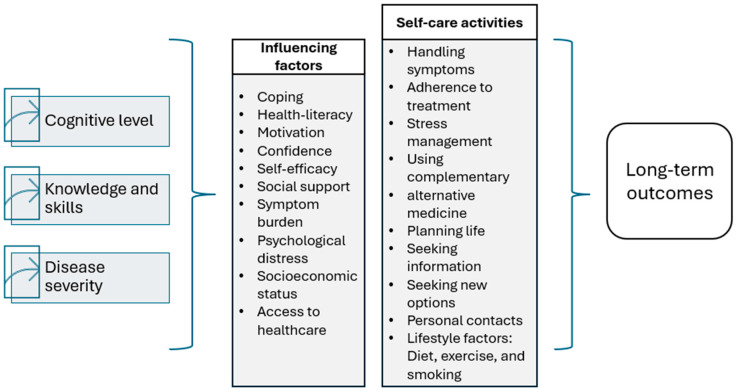
Factors to be included for long-term outcomes.

In research there are conceptual models depicting the relationship between self-management interventions, self-efficacy, and long-term outcomes for patients with IBD [14]. Further discussions are needed. Factors to be included in the development of long-term outcomes are provided for further discussion in the area. Future research should encompass opportunities for patients with IBD to find strategies to enhance their good health-related quality of life. In IBD, knowledge regarding the best strategies of self-management interventions is emerging yet unclear [39]. See suggested factors included for long-term outcomes (Figure 2).

### Strengths and Limitations

This research study has some limitations as a cross-sectional study and cannot establish causal relationships. The questionnaires used in this report were distributed alongside several other questionnaires. Patients might have found it too time-consuming to complete all the questions at once, leading to the returning of incomplete forms, dropouts, or not all questionnaires being answered simultaneously. The lack of objective clinical data—such as biomarkers, treatment status, or disease severity—makes it harder to contextualize the findings in terms of patient stability. However, symptom burden was expressed in the results. Several limitations should be acknowledged. Measurement constraints may affect the precision and validity of the constructs assessed, particularly in self-reported data. Sample homogeneity, such as limited diversity in demographic or clinical characteristics, may restrict the generalizability of the findings. Also, variations in the duration of illness among participants could influence coping mechanisms, perceived social support, and health-related quality of life, potentially confounding the interpretation of long-term outcomes. The response rate was approximately 46%, which seems to be adequate. This was a paper survey, and online surveys seem to have lower response rates [46]. However, the sample size of 206 patients is comparable to those in similar studies involving patients with IBD. A dropout analysis was not feasible in this data collection since the questionnaires were distributed by a third party and returned anonymously to the author, and it was not possible to send out reminders.

The number of patients participating in this research was relatively large, strengthening generalizability. Even though this is a multicenter study, the geographic area is limited, so generalizability to a broader geographic area cannot be certain. The gender distribution was 53% female in the full population, 52% female in the group with Crohn’s disease, and 54% female in the group with ulcerative colitis. Since there is no clear dominance between gender and IBD, the generalizability of this study is strengthened. The ages in the population were relatively evenly distributed between the age groups 20–29 (16.5%), 30–39 (19.9%), 40–49 (14.1%), 50–59 (13.1%), 60–69 (16.5%), and 70–79 (11.7%). However, the age groups 18–19 and 80–89 only contained 0.5% and 1.0% of the population, respectively. Considering that the disease commonly debuts at young ages, a larger sample from the group 18–19 would be more representative, since the age span is smaller in this group.

Despite the limitations, this study provides insights into coping, social support, and health-related quality of life in patients’ daily life. The findings contribute to clinical and empirical knowledge about factors affecting long-term outcomes for patients with IBD.

## 5. Clinical Implications

Greater attention on factors affecting long-term outcomes in patients with IBD has been demonstrated by this study. Effective management of patients with IBD should include both medical and surgical treatment and support for coping strategies, integrating psychological and social support into the care plans for patients with IBD. Interventions in individual needs, considering gender differences and personal support systems, can improve long-term outcomes. The holistic approach can enhance quality of life and long-term health outcomes.

## 6. Conclusions

This study highlights the role of gender in emotional support, with differences observed in having someone one feels close to and the size of one’s social network. Additionally, social support, particularly having someone at home or to provide comfort, was positively associated with well-being. Active coping (problem-focused coping) is used among patients with IBD in this study. Social support and coping are important factors when discussing factors affecting long-term outcomes and self-care compliance. Well-being was positively linked to coping behaviors, such as reframing situations positively to find strategies, as well as negative aspects with negative emotions and alcohol use, suggesting that symptom management plays an important role in overall well-being. Worry was linked to maladaptive coping, such as giving up, and was associated with symptom burden and bowel interference. Interestingly, no significant correlations were found between symptom burden and coping or social support, indicating the complexity of these relationships. These findings underscore the importance of psychosocial interventions to enhance coping and support mechanisms in improving health-related quality of life for patients with IBD.

## Figures and Tables

**Table 1:** Items presented for each of the facets in the Brief Cope [30].

- Active coping, (Problem-Focused) - Use of informational support, (Problem-Focused) - Positive reframing, (Problem-Focused) - Planning, (Problem-Focused) - Emotional support, (Emotion-Focused) - Venting, (Emotion-Focused) - Humor, (Emotion-Focused) - Acceptance, (Emotion-Focused) - Religion, (Emotion-Focused) - Self-blame, (Emotion-Focused) - Self-distraction, (Avoidant) - Denial, (Avoidant) - Substance use, (Avoidant) - Behavioral disengagement, (Avoidant)	

**Table 2:** Items presented for the modified form of the Availability and attachment scale [34].

Variables	Response
Is there a particular person you feel you can truly rely on for support?	No/yes, but I don’t need/yes
2. Is there a particular person you feel very close to?	no/not sure/yes
3. Do you have a special person with whom you can share your feelings when you feel happy?	no/yes
4. Do you have someone with whom you can share your innermost feelings and confide in?	no/yes
5. Does it happen that someone holds you for comfort or support?	no/yes
6. Do you think the people at home or others truly appreciate what you do for them? Yes, not enough, not at all	Yes, not enough, not at all
7. How many people do you know and stay in contact with who share your interests	none/1–2/3–5/6–10/11–15/more than 15

**Table 3:** Patient characteristics.

Variables	CD	UC	Total
Number of patients, n (%)	77 (37.4)	129 (62.6)	206
Age, median (years/range)	49 (19–87)	48 (20–83)	48 (19–87)
Time since diagnosis, median (years/range)	14 (0–50)	10 (0–50)	12 (0–50)
Female/male ratio	40:37	70:59	110:96
Surgical procedure, n (%)	42 (54.5)	13 (10)	55 (27%)
Missing data age (n = 9), time since diagnosis (n = 4)			

**Table 4:** Health-related quality of life [35].

**Items in Short Health Scale**	**CD**	**UC**
How severe symptoms do you suffer from your bowel disease?	23 (48)	22 (39.5)
Does your bowel disease interfere with your activities in daily life?	29 (58.2)	19 (50.5)
How much worry does your bowel disease cause?	36 (51.7)	31.5 (54.5)
What is your general sense of well-being?	28 (42.2)	22.5 (41.2)

Presented in millimeters as median and IQR. 0 = no symptoms, no interference with daily function, no disease-related worry, and a high sense of well-being. 100 = Very severe symptoms, very high degree of interference with daily life, constant disease-related worry, and a dreadful sense of well-being.

**Table 5:** Top 10 coping strategies.

Item	M*	SD**	Md***
Item 2. I’ve been concentrating my efforts on doing something about the situation I’m in.	2.63	0.84	3.00
Item 5. I’ve been getting emotional support from others.	2.63	0.88	3.00
Item 7 I’ve been taking action to try to make the situation better.	2.76	0.87	3.00
Item 12. I’ve been trying to see it in a different light, to make it seem more positive.	2.42	0.82	2.00
Item 14. I’ve been trying to come up with a strategy about what to do.	2.78	0.86	3.00
Item 15. I’ve been getting comfort and understanding from someone.	2.62	0.84	2.00
Item 17. I’ve been looking for something good in what is happening.	2.63	0.82	3.00
Item 23. I’ve been trying to get advice or help from other people about what to do.	2.33	0.75	3.00
Item 24. I’ve been learning to live with it.	2.95	0.82	3.00
Item 25. I’ve been thinking hard about what steps to take.	2.62	0.86	3.00

M* = mean; SD** = standard deviation; Md*** = median.

**Table 6:** Coping and well-being.

Coping Variables	Well-Being (Rho)	*p*-Value
I use alcohol or other drugs to feel better	0.167	0.018
I use alcohol or other drugs to help me get through it	0.149	0.035
I try to look at it in a different light to make it seem more positive	0.188	0.008
I try to come up with a strategy for what I should do	0.192	0.009
I joke about it	0.152	0.033
Negative emotions	0.182	0.011

## Data Availability

Data are contained within the article.

## Public Involvement Statement

There was no public involvement in any aspect of this research. The patients were recruited from gastroenterology clinics.

## Guidelines and Standards Statement

This manuscript was drafted in accordance with the STROBE guidelines for reporting observational studies.

## Abbreviations

The following abbreviations are used in this manuscript:

IBD	Inflammatory Bowel Disease
UC	Ulcerative Colitis
CD	Crohn´s Disease
